# Serum non-high-density lipoprotein cholesterol predicts distant metastases following resection of stages I to III colorectal cancer

**DOI:** 10.1097/MD.0000000000040172

**Published:** 2024-10-18

**Authors:** Ronghua Fang, Aijun Shi, Hui Cong, Xiuying Shi

**Affiliations:** a Department of Laboratory Medicine, Affiliated Hospital of Nantong University, Nantong City, Jiangsu Province, China; b Medical school of Nantong University, Nantong City, Jiangsu Province, China; c Department of Laboratory Medicine, Rudong Hospital of Traditional Chinese Medicine, Nantong City, Jiangsu Province, China; d Department of Blood Transfusion, Affiliated Hospital of Nantong University, Nantong City, Jiangsu Province, China.

**Keywords:** colorectal cancer, non-high-density lipoprotein cholesterol, distant metastasis

## Abstract

The aim of this study was to examine the relationship between levels of non-high-density lipoprotein cholesterol (non-HDL-C) and postoperative distant metastasis for stages I to III colorectal cancer (CRC). Demographic, clinicopathological, and lipid data were collected from 588 patients, who were subsequently grouped according to their non-HDL-C levels. The primary endpoint was distant metastasis, survival without distant metastasis-free survival (DMFS). The association between non-HDL-C and pathological features, as well as postoperative distant metastasis, was assessed using a chi-square test, Mann–Whitney U test, and Cox proportional hazard regression model. The correlation between DMFS and non-HDL-C levels was analyzed employing the Kaplan–Meier method and log-rank test. The incidence of postoperative distant metastasis was significantly higher in the high non-HDL-C group (34.8%) compared to the low non-HDL-C group (18.2%) (*P* < .001). Non-HDL-C levels were significantly higher in the metastasis group than in the nonmetastasis group (*P* = .001). Multivariate Cox proportional hazards identified non-HDL-C ≥ 4.1mmol/L(HR: 2.604; 95% CI: 1.584–4.282; *P* = .001) as independent risk factors for postoperative distant metastasis. The high non-HDL-C group exhibited a higher rate of distant metastasis and a shorter duration of DMFS (HR: 2.133; 95% CI: 1.404–3.240; *P* < .001). Our study suggests that high levels of non-HDL-C (≥4.1 mmol/L) may potentially serve as predictors for postoperative distant metastasis in stages I to III CRC.

## 1. Introduction

Colorectal cancer (CRC) is a prevalent gastrointestinal malignancy, and its incidence and mortality rates have gradually increased in recent years. Projections for 2020 indicate that CRC accounts for approximately 10% of new cancer cases and 9.4% of deaths, underscoring its significant role in the global burden of cancer.^[1]^ Despite the majority of patients undergoing radical surgery, it is concerning that recurrence or metastasis rates remain as high as 35% to 45% within 5 years. This elevated rate not only impacts patients’ quality of life but also exacerbates disease-related mortality.^[2,3]^ Particularly for individuals with metastatic CRC, prognosis tends to be poor with relatively high mortality rates. Therefore, identifying and effectively managing risk factors for distant metastases of CRC is critical.

The postoperative progression of CRC is influenced by many factors. Histopathology features were closely related to recurrence, metastasis and prognosis. Studies have confirmed that factors such as TNM stage, number of positive lymph nodes, vascular cancer embolism, peripheral nerve invasion, and degree of differentiation have significant effects on postoperative metastasis prognosis of CRC patients.^[4–6]^

Lipids play a crucial role in various biological processes, encompassing cell proliferation, differentiation, apoptosis, inflammation, membrane homeostasis, chemotherapy response, and drug resistance. Moreover, dyslipidemia has been firmly associated with tumor occurrence and development.^[7]^ Its association with the risk of CRC has also been documented.^[8]^ However, the relationship between different lipid types and postoperative metastasis of CRC remains uncertain.^[9]^

Conventional blood lipid detection indicators primarily include triglycerides (TG), total cholesterol (TC), low-density lipoprotein cholesterol (LDL-C), and high-density lipoprotein cholesterol (HDL-C). Non-high-density lipoprotein cholesterol (non-HDL-C) refers to all forms of cholesterol except HDL-C, including very low-density lipoprotein cholesterol (VLDL-C), intermediate-density lipoprotein cholesterol (IDL-C), LDL-C, and lipoprotein(a). Non-HDL-C has been found to have superior predictive capability for atherosclerotic cardiovascular disease (ASCVD) risk compared to LDL-C.^[10–12]^ The “common soil hypothesis” suggests a potential correlation or interaction mechanism between cardiovascular disease and tumors.^[13]^ Therefore, we propose a daring speculation that non-HDL-C may be involved in the occurrence and progression of CRC.

Limited research has been conducted on the association between non-HDL-C and tumors. For instance, increased levels of non-HDL-C have been found to be correlated with a higher risk of all-cause mortality from obesity-related cancers,^[14]^ while a high ratio of non-HDL-C/HDL-C has been linked to poorly differentiated or high-grade pancreatic neuroendocrine tumors.^[15]^ However, no studies have reported on the correlation between non-HDL-C levels and the progression of colorectal tumors to date. The aim of this study was to examine the correlation between non-HDL-C levels and pathological characteristics, particularly postoperative distant metastases, in patients diagnosed with CRC.

## 2. Materials and methods

### 2.1. Study population

The sample of this study was 588 patients who underwent surgery and were pathologically confirmed in the Affiliated Hospital of Nantong University from February 2013 to December 2017, including 378 males and 210 females. The median age of patients was 63 years, and the interquartile interval (IQR) was 56-71 years. The inclusion criteria consisted of the following: patients undergoing radical surgery; histopathology confirmation of CRC; and absence of distant metastasis confirmed by imaging or pathology. The exclusion criteria included: a history of other malignancies; a history of ASCVD; and insufficient important index data and follow-up information. Ethical approval was obtained from the Ethics Committee at the Affiliated Hospital of Nantong University, under protocol number 2022-K057-01.

### 2.2. Postoperative distant metastasis

By December 2022, distant metastasis had occurred in a total of 130 patients (22.1%) following surgery. Among the group with metastasis, single-site metastasis was observed in 90 patients (69.2%), while multi-site metastasis occurred in 40 patients (30.8%). The lung was found to be the most common site of metastasis, with 60 cases, followed by the liver with 56 cases, peritoneum and pelvic cavity with 34 cases, and bone with 18 cases.

### 2.3. Data collection

TC and HDL-C data were collected in the week preceding surgery. Non-HDL-C was calculated by subtracting HDL-C from TC. According to the Chinese guideline for lipid management (2023), subjects were categorized into 2 groups based on a non-HDL-C threshold of 4.1 mmol/L.^[16]^ Information such as age, gender, and pathological data were also gathered for each patient enrolled in the study. The tumor stage was determined using the 8th edition of the American Joint Committee on Cancer TNM staging system.^[17]^

### 2.4. Follow-up

Follow-up assessments were conducted at 3, 6, and 12 months postsurgery, with subsequent annual evaluations. Distant metastases were identified through imaging techniques such as magnetic resonance imaging (MRI) or computed tomography (CT), which were subsequently confirmed by histological analysis. The time interval between radical surgery and the diagnosis of distant metastasis was defined as the metastasis time. The outcome variables included distant metastasis or death, as well as distant metastasis-free survival (DMFS). The last follow-up was conducted in December 2022.

### 2.5. Statistical analysis

Statistical analysis was conducted using SPSS 20.0 (IBM Corp., New York, NY, USA), and data visualization was performed with GraphPad Prism 7.0 (GraphPad Software Inc., San Diego, CA, USA). Categorical variables were presented as numbers (%) and group differences were assessed using the chi-square test. Non-normally distributed continuous variables were reported as median and IQR, and differences between groups were tested using the Mann–Whitney *U* test. The Cox proportional hazards model was used to analyze the association between factors and distant metastasis. The Kaplan–Meier method and log-rank test were employed to examine the correlation between non-HDL-C levels and 5-year DMFS. Statistical significance was defined as *P* < .05.

## 3. Results

### 3.1. Clinicopathological features of CRC patients with different non-HDL-C group

Table 1 presents a summary of the clinicopathological features of CRC patients in different non-HDL-C level groups. Significant disparities were observed between the 2 groups in terms of tumor site, N stage, TNM stage, and perineural infiltration (*P < *.05 for all). The high non-HDL-C group exhibited a significantly higher incidence of postoperative distant metastasis (34.8%) compared to the low non-HDL-C group (18.2%) (*P < *.001).

**Table 1:** Clinicopathological features of CRC patients with different non-HDL-C groups.

Characteristics	Low non-HDL-C	High non-HDL-C	*χ* ^2^	*P* value
	<4.1 mmol/L (N = 450)	≥4.1 mmol/L (N = 138)		
Gender				
Male (%)	298 (66.2)	80 (58.0)	3.132	.077
Female (%)	152 (33.8)	58 (42.0)		
Age (yr)				
<60 (%)	162 (36.0)	56 (40.6)	0.949	.330
≥60 (%)	288 (64.0)	82 (59.4)		
Tumor size (cm)				
<5 (%)	384 (85.3)	120 (87.0)	0.227	.634
≥5 (%)	66 (14.7)	18 (13.0)		
Primary site				
Left colon (%)	116 (25.8)	20 (14.5)	7.565	.006
Right colon (%)	334 (74.2)	118 (85.5)		
Differentiation				
Well and moderately (%)	412 (91.6)	130 (94.2)	1.026	.311
Poorly (%)	38 (8.4)	8 (5.8)		
T stage				
T_1-2_ (%)	106 (23.6)	22 (15.9)	3.595	.058
T_3-4_ (%)	344 (76.4)	116 (84.1)		
N stage				
N_0_ (%)	274 (60.9)	58 (42)	15.282	<.001
N_1-2_ (%)	176 (39.1)	80 (58)		
TNM stage				
I to II (%)	246 (54.7)	54 (39.1)	10.201	.001
III (%)	204 (45.3)	84 (60.9)		
Perineural invasion				
Negative (%)	341 (75.8)	91 (65.9)	5.242	.022
Positive (%)	109 (24.2)	47 (34.1)		
Vascular invasion				
Negative (%)	259 (57.6)	71 (51.4)	1.599	.206
Positive (%)	191 (42.4)	67 (48.6)		
Distant metastasis				
Negative (%)	368 (81.8)	90 (65.2)	16.819	<.001
Positive (%)	82 (18.2)	48 (34.8)		

Numbers (percentages) represent categorical variables: *P* value was determined using a Chi-square test.

Non-HDL-C = non-high-density lipoprotein, TNM = tumor node metastasis.

### 3.2. Correlation of non-HDL and pathological features with distant metastasis

Table 2 presents a comparison between the distant metastasis group and the non-distant metastasis group, highlighting significant differences in tumor site, N stage, TNM stage, and vascular invasion (*P* < .05 for all). Additionally, the distant metastasis group exhibited significantly higher levels of non-HDL-C compared to the nonmetastasis group (*P* = .001)

**Table 2:** Correlation of non-HDL and pathological features with distant metastasis.

Characteristics	Overall	Nonmetastasis	Metastasis	*χ*^2^/Z	*P* value
	(N = 588)	(N = 458)	(N = 130)		
Gender					
Male (%)	378 (64.3)	288 (62.9)	90 (69.2)	1.778	.182
Female (%)	210 (35.7)	170 (37.1)	40 (30.8)		
Age (yr)					
<60 (%)	218 (37.1)	174 (38.0)	44 (33.8)	0.746	.388
≥60 (%)	370 (62.9)	284 (62.0)	86 (66.2)		
Tumor size (cm)					
<5 (%)	504 (85.7)	390 (85.2)	114 (87.7)	0.533	.465
≥5 (%)	84 (14.3)	68 (14.8)	16 (12.3)		
Primary site					
Left colon (%)	136 (23.1)	120 (26.2)	16 (12.3)	10.993	.001
Right colon (%)	452 (76.9)	338 (73.8)	114 (87.7)		
Differentiation					
Well and moderately (%)	542 (92.2)	420 (91.7)	122 (93.8)	0.645a	.422
Poorly (%)	46 (7.8)	38 (8.3)	8 (6.2)		
T stage					
T_1-2_ (%)	128 (21.8)	104 (22.7)	24 (18.5)	1.072a	.301
T_3-4_ (%)	460 (78.2)	354 (77.3)	106 (81.5)		
N stage					
N_0_ (%)	332 (56.5)	272 (59.4)	60 (46.2)	7.215	.007
N_1-2_ (%)	256 (43.5)	186 (40.6)	70 (53.8)		
TNM stage					
I to II (%)	300 (51.0)	246 (53.7)	54 (41.5)	6.005	.014
III (%)	288 (49.0)	212 (46.3)	76 (58.5)		
Perineural invasion					
Negative (%)	430 (73.1)	340 (74.2)	90 (69.2)	1.291	.256
Positive (%)	158 (26.9)	118 (25.8)	40 (30.8)		
Vascular invasion					
Negative (%)	328 (55.8)	286 (62.4)	42 (32.3)	37.29	<.001
Positive (%)	260 (44.2)	172 (37.6)	88 (67.7)		
HDL-C (mmol/L)	1.18 (0.98–1.44)	1.18 (0.98–1.43)	1.27 (0.95–1.46)	0.729	.466
Non-HDL-C (mmol/L)	3.47 (2.87–4.01)	3.45 (2.81–3.93)	3.59 (2.95–4.41)	3.28	.001

Median and interquartile intervals (IQR) for continuous variables: *P* value determined using the Mann–Whitney *U* test. Numbers (percentages) for categorical variables: *P* value was determined using a Chi-square test.

HDL-C = high-density lipoprotein cholesterol, non-HDL-C = non-high-density lipoprotein cholesterol, TNM = tumor node metastasis.

### 3.3. Univariate and multifactorial analysis of factors associated with distant metastasis

The factors associated with distant metastasis were analyzed through univariate and multifactorial analysis, and the results are summarized in Table 3. The findings from the univariate Cox analyses indicate a significant association between postoperative distant metastases and several factors: right colon, N_1-2_, TNM stage III, vascular invasion, and non-HDL-C ≥ 4.1mmol/L (*P < *.05 for all). In the multivariate Cox proportional hazards model, vascular invasion (HR: 2.311; 95% CI: 1.254–4.259; *P* = .007) and non-HDL-C ≥ 4.1mmol/L (HR: 2.604; 95% CI: 1.584–4.282; *P* = .001) were identified as independent risk factors for distant metastasis.

**Table 3:** Univariate and multivariate analyses of factors associated with distant metastasis.

Characteristics	Univariate analysis			Multivariate analysis		
	HR	95% CI	*P* value	HR	95% CI	*P* value
Gender						
Male	1		.194			
Female	1.280	0.882 to 1.857				
Age (years)						
<60	1		.404			
≥60	1.167	0.812 to 1.679				
Tumor size (cm)						
<5	1		.632			
≥5	0.880	0.521 to 1.485				
Primary site						
Left colon	1		.002	1		
Right colon	2.325	1.377 to 3.923		1.326	0.619 to 2.844	.468
Differentiation						
Well and moderately	1		.403			
Poorly	0.737	0.361 to 1.507				
T stage						
T_1-2_	1		.256			
T_3-4_	1.293	0.83 to 2.013				
N stage						
N_0_	1		.008	1		.145
N_1-2_	1.592	1.127 to 2.247		1.576	0.855 to 2.903	
TNM stage						
I to II	1		.017	1		.407
III	1.53	1.080 to 2.169		1.29	0.706 to 2.357	
Perineural invasion						
Negative	1		.558			
Positive	1.170	0.692 to 1.978				
Vascular invasion						
Negative	1		<.001	1		.007
Positive	3.022	1.817 to 5.024		2.311	1.254 to 4.259	
Non-HDL-C (mmol/L)						
< 4.1	1		<.001	1		.001
≥ 4.1	2.134	1.494 to 3.048		2.604	1.584 to 4.282	

CI = confidence interval, HR = hazard ratio.

### 3.4. Correlation analysis of non-HDL-C levels and the 5-year DMFS

**Figure 1.**
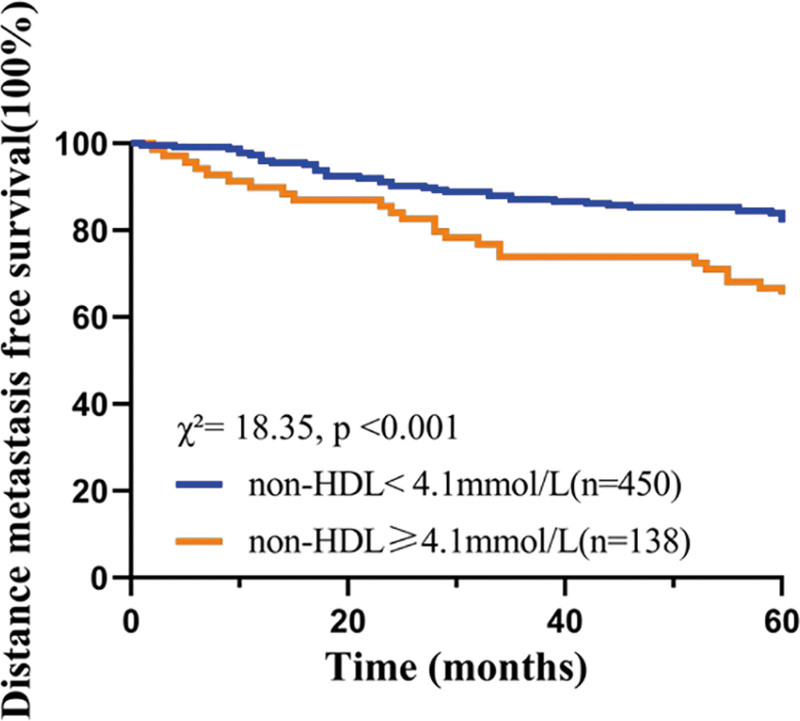
Five-year distant metastasis-free survival according to different levels of non-HDL-C in CRC patients.

The Kaplan–Meier analysis revealed a statistically significant difference in 5-year DMFS between the non-HDL-C ≥ 4.1mmol/L group and the non-HDL-C < 4.1mmol/L group, with patients in the former exhibiting a higher rate of distant metastasis and shorter DMFS duration. The corresponding Kaplan–Meier curves are depicted in Figure 1.

## 4. Discussion

Lipids play crucial roles in various biological functions, such as maintaining cell membrane integrity, storing energy, transmitting signals, and synthesizing hormones. Maintaining lipid homeostasis is essential for optimal physiological function and overall well-being. However, excessive lipid accumulation has the potential to facilitate tumor cell formation.^[18,19]^ In recent years, there has been an increasing amount of research conducted to explore the correlation between dyslipidemia and tumors.^[20,21]^ One study demonstrated that a score combining several measures including TG and HDL could predict the risk of advanced colorectal tumors.^[22]^ Another study found that cholesterol may contribute to tumor development by promoting excessive division and proliferation of intestinal stem cells in vivo.^[23]^ However, several studies have yielded contrasting findings. For instance, serum TC was found to have a negative correlation with overall cancer risk in females.^[24]^ Another study demonstrated no significant association between lipids and CRC risk after adjusting for obesity.^[25]^ Consequently, further investigation into the precise relationship between dyslipidemia and the progression of CRC is greatly needed. Given the established link between non-HDL-C and ASCVD, as well as the shared risk factors and “common soil hypothesis” between ASCVD and CRC,^[26–28]^ our study aims to investigate the association between non-HDL-C levels and postoperative metastasis of CRC.

Metastasis, which denotes the dissemination of malignant cells to distant organs, is influenced by factors including tumor type and grade, invasive potential of cancer cells, immunological competence, and a variety of other factors.^[29–31]^ Our study revealed a significant correlation between elevated non-HDL-C levels and an increased risk of postoperative distant metastases in CRC patients. Patients with distant metastasis demonstrated notably higher non-HDL-C levels compared to those without, suggesting a heightened susceptibility to this adverse outcome. Furthermore, these individuals experienced a shorter DMFS. As a vital component of blood lipid, increased non-HDL-C plays an important role in cancer proincreased non-HDL-C, as a crucial component of blood lipid, plays a significant role in the progression of cancer. This phenomenon can be explained through several potential mechanisms. Firstly, tumor cells hinder the conversion of VLDL and LDL, leading to elevated levels of non-HDL-C. This creates a fatty tumor microenvironment that promotes the proliferation of cancer cells.^[32]^ Secondly, in nutrient-depleted tumor microenvironments or distant tissues after metastasis, increased non-HDL-C alleviates cellular stress responses and confers resistance to chemotherapy or radiation therapy.^[33]^ In addition, the increase in non-HDL-C is associated with the secretion of angiogenic factors and the activation of the angiogenic process, which promote the transformation of cancer cells into an aggressive phenotype.^[34,35]^ These mechanisms collectively create an environment that promotes the growth and infiltration of cancer cells. Although further investigation is needed to fully understand the underlying mechanism, our research highlights the clinical significance of non-HDL-C and suggests a more proactive postoperative follow-up for patients with elevated preoperative levels of non-HDL-C.

Our study revealed a significantly higher prevalence of advanced TNM stage and lymph node metastasis in the high non-HDL-C group. Furthermore, there was a significantly greater proportion of patients with advanced TNM stage and lymph node metastasis in the metastatic group. These findings are consistent with previous studies that have shown a correlation between lipid levels and pathological features of CRC, including lymph node metastasis and TNM stage.^[36]^ For instance, Zhang et al^[37]^ found an association between TC and TG levels with TNM staging in CRC patients. Similarly, the study conducted by Sako et al^[38]^ revealed that patients suffering from esophageal cancer with lymph node metastasis exhibited notably elevated levels of TC and TG in comparison to those without such metastasis. This further supports the notion that elevated non-HDL-C levels may be linked to malignant progression and unfavorable prognosis in CRC.

Furthermore, numerous studies have consistently shown a significant correlation between vascular invasion and early recurrence as well as distant metastasis in patients with CRC.^[39]^ A study indicated that individuals with extramural invasion had lower overall survival rates and disease-free survival rates compared to those without invasion or with intramural invasion.^[40]^ Although the specific location of vascular invasion (intramural or extramural) was not identified in our study, our findings strongly suggest that vascular invasion is an independent risk factor associated with distant metastases following CRC surgery.

Our practical retrospective analysis confirms the correlation between non-HDL-C and the pathological characteristics as well as distant metastasis of CRC. The non-HDL-C data can be easily obtained through a simple calculation, without imposing any additional financial burden on patients. Furthermore, non-HDL-C remains stable in serum and is minimally affected by TG levels and food intake, demonstrating its significant clinical applicability.

## 5. Conclusion

In conclusion, this study demonstrates a significant correlation between non-HDL-C levels and distant metastasis in stages I to III CRC. It indicates that patients with elevated non-HDL-C (≥ 4.1mmol/L) are at a higher risk of developing distant metastases and have shorter durations of DMFS. These findings offer valuable insights into the prognosis of CRC and can potentially assist physicians in optimizing postsurgical follow-up monitoring.

## 6. Limitation

Due to the potential influence of confounding factors in retrospective studies, this study may not accurately estimate the true association between non-HDL-C levels and CRC metastasis, as other risk factors such as genetic predisposition and lifestyle also impact. However, our study highlights a significant finding that patients with high non-HDL-C exhibited a higher rate of postoperative metastasis compared to those with lower levels. Furthermore, non-HDL-C was significantly elevated in the postoperative metastasis group compared to the nonmetastatic group. Additionally, since our research was conducted in a single center, it is desirable to conduct multi-center and prospective studies in the future.

## Author contributions

**Conceptualization:** Ronghua Fang, Aijun Shi, Hui Cong, Xiuying Shi.

**Data curation:** Ronghua Fang, Aijun Shi, Hui Cong, Xiuying Shi.

**Formal analysis:** Ronghua Fang, Aijun Shi.

**Investigation:** Ronghua Fang, Aijun Shi.

**Methodology:** Ronghua Fang, Aijun Shi.

**Project administration:** Hui Cong, Xiuying Shi.

**Validation:** Ronghua Fang.

**Writing – original draft:** Ronghua Fang, Aijun Shi.

**Writing – review & editing:** Ronghua Fang.
